# Effect of the Second Phases on Composite Spinning-Extrusion Forming and Mechanical Properties of Al–Cu–Li Alloy

**DOI:** 10.3390/ma16093573

**Published:** 2023-05-06

**Authors:** Huaqiang Zeng, Dongfeng Shi, Ying Zheng, Jin Zhang

**Affiliations:** 1 Light Alloy Research Institute, Central South University, Changsha 410083, China; zenghuaqiang163@163.com (H.Z.); dongfeng.shi@csu.edu.cn (D.S.); zhangjinlari@csu.edu.cn (J.Z.); 2 State Key Laboratory of Precision Manufacturing for Extreme Service Performance, Central South University, Changsha 410083, China

**Keywords:** Al–Cu–Li alloy, composite spinning-extrusion forming, second phases, cylindrical parts with ribs, mechanical properties, microstructure

## Abstract

The aim of this work is to investigate the effect of different second phases on the composite spinning-extrusion forming and mechanical properties of Al–Cu–Li alloy. With that purpose, four kinds of second phases blanks were controlled using preheating treatment, composite spinning-extrusion forming and mechanical properties test. Then, the correlation between the second phases and mechanical properties was further analyzed using electron backscattered diffraction and transmission electron microscopy. The results indicated that different second phases of Al–Cu–Li alloy can be regulated via reasonable preheating treatment. In addition, different second phases in the blank have various influences on composite spinning-extrusion forming, microstructure and mechanical properties of cylindrical parts. Dissolving the coarse second phases particles and precipitating the Al_3_Zr dispersoid in the blank can effectively improve the composite spinning-extrusion forming, inhibit the abnormal growth of recrystallized grains, and significantly enhance the mechanical properties of cylindrical parts with ribs. After regulation, the average grain size of the cylindrical parts is refined from about 90 μm to about 45 μm, and the average diameter of T_1_ phase is refined from 107 nm to 77 nm. In addition, the ultimate tensile strength, yield strength and elongation of cylindrical parts are increased from 555 MPa to 588 MPa, 530 MPa to 564 MPa, and 9.1% to 11%, respectively.

## 1. Introduction

Al–Cu–Li alloys are of great interest to the aerospace field owing to a number of key advantages they offer over traditional aluminum alloy [1,2,3]. Their excellent fatigue performance, lower density, and combination of higher elastic modulus and high specific strength, can lead to significant weight savings [4]. In addition, Al–Cu–Li alloy can effectively improve the carrying capacity of launch vehicles by replacing the traditional 2xxx series aluminum alloys when making the structure of the storage tanks, thereby highlighting their considerable application potential in new aerospace structures designs [5,6].

At present, the integral forming technology of lightweight structural parts has become the key to improving the lightweight level of large space equipment [7]. As an advanced integral forming technology [8], composite spinning-extrusion forming is expected to realize integral manufacturing of high-performance cylindrical parts with ribs by combining with light weight and high strength Al–Cu–Li alloy [9,10,11]. However, composite spinning-extrusion forming is a complicated process. The quality of the formed part is not only affected by the forming process parameters but also closely related to the microstructure of the material. In recent years, people have carried out extensive exploration on the integral forming process of cylinder parts with ribs by combining numerical simulation with experiments. It is found that the filling of ribs is mainly determined by material accumulation and material flow direction before rollers [12]. Optimizing the design of ribs structure [13,14] and improving the thinning rate, blank thickness and feed ratio are conducive to improving the height of ribs [15]. In addition, the integral forming of aluminum alloy cylinder parts with ribs has a unique material flow behavior and heterogeneous microstructure induced by deformation gradient [16]. Then, combined with the corresponding annealing treatment, more uniform mechanical properties can be obtained [17]. However, most of these researches focus on the influence of the forming process and the optimization of forming quality, and pay limited attention to the structure regulation of forming blank. In particular, blank microstructure is the basis of forming, and its microstructure compatibility has a crucial effect on the mechanical properties of forming parts. Therefore, it is of great significance to control the microstructure of blank formed by compound spinning-extrusion forming.

The blank microstructure of Al–Cu–Li alloy mainly consists of the grain and the second phases, among which the regulation of the second phases is the primary method to improve the properties of the alloy [18]. Therefore, a lot of studies have been conducted on the precipitation and strengthening of the second phases of Al–Cu–Li alloy [19,20,21], and the corresponding time–temperature–precipitation (TTP) curve is described [22]. However, the second phases of Al–Cu–Li alloy are complex and varied, and its changes under different thermal deformation conditions are more complex. The complex precipitation of these second phases will not only further contribute to the strength anisotropy [23,24], but also affect the quality and mechanical properties of hot forming [25,26,27]. However, the effect of the second phases of Al–Cu–Li alloy and its combination on the composite spinning-extrusion forming are still unclear, which may have a profound influence on the integral manufacturing of the cylindrical parts with ribs.

The aim of the present study is to reveal the effect of different second phases on the composite spinning-extrusion forming and mechanical properties of Al–Cu–Li alloy. To address this complex issue, four typical second phases which may exist in hot forming were controlled via preheating treatment. Then, the correlation between the second phases and the mechanical properties of the cylinder was analyzed using electron back diffraction (EBSD) and transmission electron microscopy (TEM). Finally, the optimum heat treatment process of the second phases of the Al–Cu–Li alloy blank formed by composite spinning-extrusion forming was determined. This study provided theoretical guidance for microstructure regulation of hot forming blank in related fields.

## 2. Materials and Experimental Design

The material used in this study was a spray-formed free forged annealed Al–Cu–Li alloy plate with the dimension of 700 × 500 × 20 mm. Table 1 showed the chemical compositions of the alloy, which were tested using the German SPECTRO BLUE SOP full spectrum direct reading plasma emission spectrometer. The free forging process was as follows: upsetting to φ650 × 130 mm at 440 °C, then three-way forging to 500 × 500 × 120 mm, and finally drawing length along the width and length direction to 80 × 700 × 700 mm, each drawing length to 20 mm. Firstly, a 700 × 500 × 20 mm rectangular sample was cut from the free forging plate, and then four φ200 × 20 mm cylindrical samples were cut from the rectangular sample. Subsequently, the four cylindrical samples were subjected to different annealing treatments. The first sample was not annealed. The second sample was water quenched after 510 °C/4 h high temperature treatment, and then annealed at 350 °C/2 h. The third sample was insulated for 12 h after rising the room temperature to 510 °C at a heating rate of 1 °C/min. Based on the same heat treatment as the third sample, the fourth sample was further water quenched, and then annealed at 400 °C/10 h. For convenience of discussion, the four samples were denoted as AN, A350, A510 and A400, respectively. Then, the samples were subjected to composite spinning-extrusion forming under the same process parameters, and finally subjected to T83 process treatment (510 °C/1 h+ water quenching + 3% pre-stretching +160 °C/27 h). Similarly, for simplicity, the four cylindrical parts with ribs after forming were denoted as PAN, PA350, PA510 and PA400, respectively.

**Figure 1:**
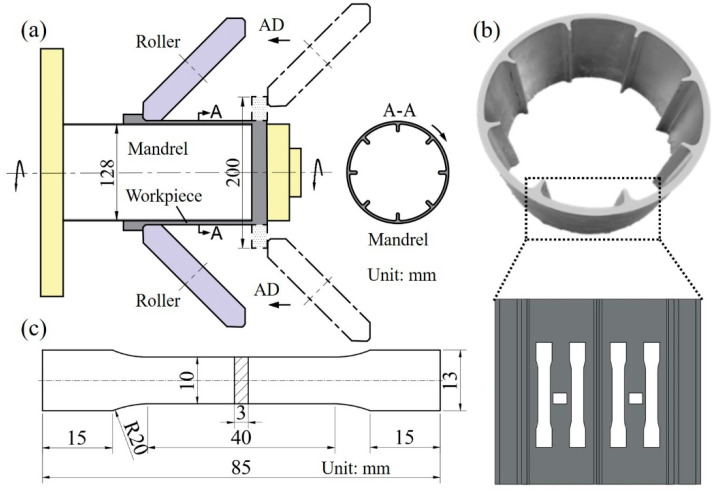
Composite spinning-extrusion forming schematic diagram and sampling diagram; (**a**) composite spinning-extrusion forming schematic diagram, (**b**) selected positions, (**c**) tensile dimension at room temperature.

The composite spinning-extrusion forming was carried out on a PS-CNCSXY1000 NC spinning machine. The forming process parameters were shown in Table 2, and the forming schematic diagram and sampling diagram were shown in Figure 1. As shown in Figure 1a, during composite spinning-extrusion forming, the rollers were fed along the axial direction (AD) in accordance with a certain track, and then acted on the blank rotating with the mandrel in the form of point-by-point high pressure to produce plastic deformation. Finally, the cylindrical part was manufactured. The total forming time was 10 min. In strict accordance with ISO 6892-1:2009, the tensile sample was cut from the cylindrical part along the axial direction. Its geometric size was shown in Figure 1c, and the sampling diagram was shown in Figure 1b. The pre-deformation and room temperature tensile tests were carried out on the CSS-44100 tensile testing machine. The tensile speed was 2 mm/min, and the elongation of the alloy was measured with a 25 mm clip-on extensometer. Five tensile tests for each state were conducted to diminish the systematical errors.

The hardness test samples and all microscopic test samples were cut from the thin wall of the cylindrical part; the sampling diagram was shown in Figure 1b. The hardness measurement was performed on a CMT-5105 microhardness tester with a load of 1000 N and a residence time of 10 s. Optical microscope (OM) samples were observed using Olympus DSX500 after mechanical grinding and polishing. The samples of scanning electron microscope (SEM) and EBSD were observed via the Zeiss Evo MA10 equipped with EBSD probe after mechanical grinding and polishing. In addition, the OM samples needed to be wiped and corroded in Keller’s reagent for 30 s before observation, and the EBSD samples electropolished in 30% nitric acid and 70% methanol at a constant voltage of 20 V for 15 s to remove the stress layer. X-ray diffractometer (XRD) measurements were made on an Advance D8 with sample sizes of 12 × 12 × 5 mm and 2θ ranges from 20° to 90°. The samples for TEM observation were mechanically thinned to about 90 μm and then punched into φ3 mm using a punching machine. Then, the samples were Twin-jet electropolished in a mixture of 20% nitric acid and 80% methanol with the electrolytic current 60mA at −30 °C Finally, the samples were observed under a FEI Titan F20 G2 with an operating voltage of 200 kV.

## 3. Results

### 3.1. Microstructure of Samples under Different Heat Treatments

**Figure 2:**
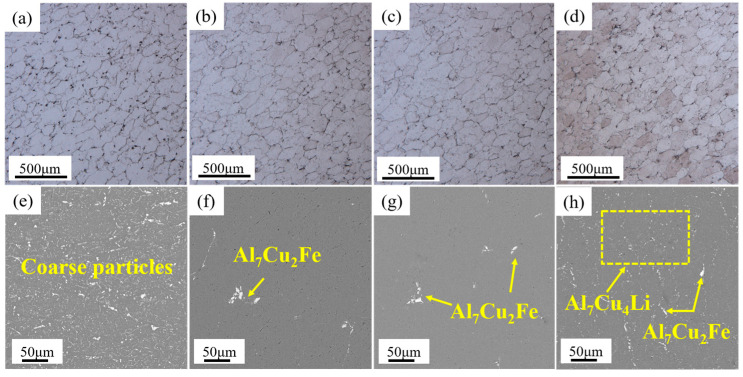
OM and SEM results of samples after different heat treatments; (**a**,**e**) AN, (**b**,**f**) A350, (**c**,**g**) A510, (**d**,**h**) A400.

**Figure 3:**
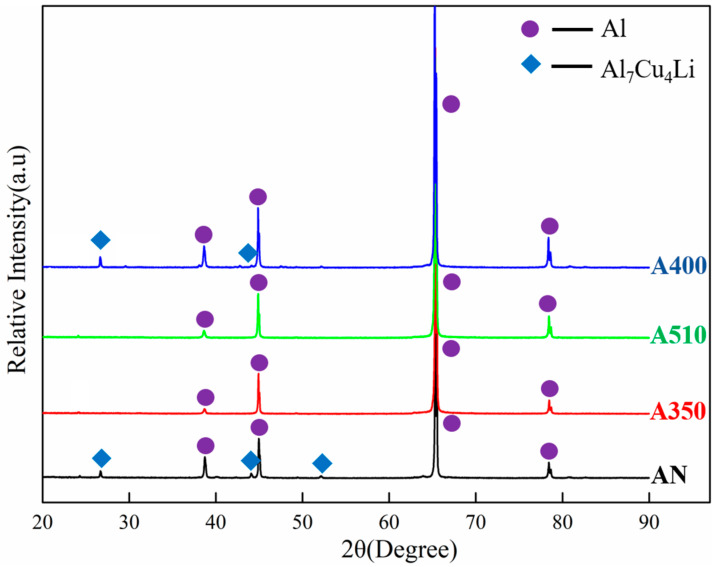
The XRD diffraction patterns of the samples.

**Figure 4:**
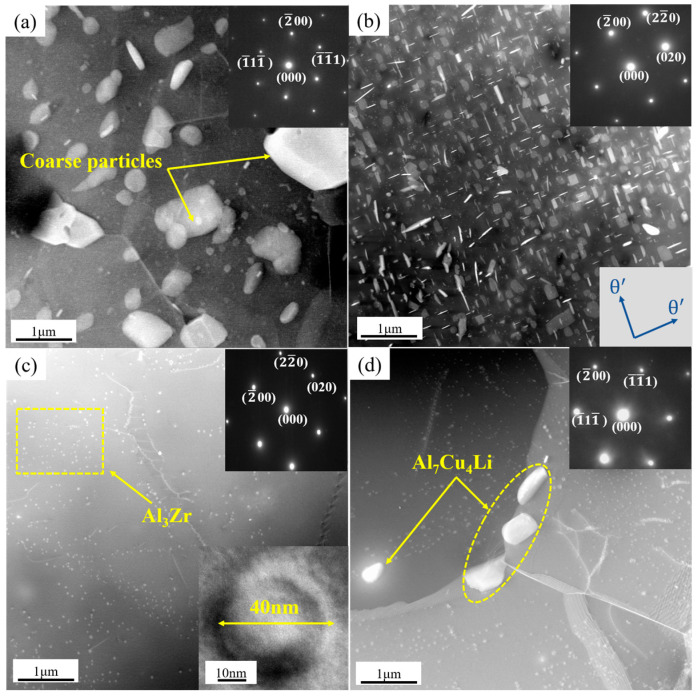
TEM results of samples after different heat treatments; (**a**) AN, (**b**) A350, (**c**) A510, (**d**) A400.

Figure 2 illustrated the OM and SEM results of the samples under different heat treatment regulation. Figure 3 depicted the XRD diffraction patterns of the samples. The OM results of Figure 2a–d showed that after a series of heat treatments, the microstructure of the spray-formed Al–Cu–Li alloy were equiaxed with an average grain size of 156 μm. It can be seen in Figure 3 that there were many Al_7_Cu_4_Li phases in the AN sample. However, after annealing at high temperature, the Al_7_Cu_4_Li phase dissolved back into the matrix. After water quenching and annealing at 400 °C/10 h, Al_7_Cu_4_Li phase was precipitated from the A400 sample. In order to determine the influence of heat treatment on the phase types in the alloy, TEM analysis was performed on the different samples, and the TEM result was shown in Figure 4.

The results of Figure 2e showed that the initial scanning microstructure of sample 1 contained a large number of coarse second phases particles. The size of these second phases particles reaches micron (as shown in Figure 4a). Through Energy Dispersive Spectrometer (EDS) and XRD analysis (as shown in Figure 3), combined with the main precipitated phase of Al–Cu–Li alloy [6,28], the second phases of AN sample could be obtained as the mixture of Al_2_Cu, Al_7_Cu_4_Li and Al_7_Cu_2_Fe phases. According to the results of Figure 2f, after heat treatment at 510 °C/4 h, most of the second phases particles in the A350 sample were dissolved back into the aluminum matrix. Based on EDS analysis, the remaining second phase particles of the A350 sample were Al_7_Cu_2_Fe. In addition, the results of Figure 4b showed that many θ’ phases were precipitated from the A350 sample during the annealing process at 350 °C/2 h. Therefore, the second phases of the A350 sample were a mixture of the θ’ and Al_7_Cu_2_Fe phases. The results of Figure 2g and Figure 4c showed that in addition to the stable Al_7_Cu_2_Fe particles in the second phases of the A510 sample heated via slope heating, Al_3_Zr dispersoid with the size of about 40 nm was precipitated. The results in Figure 2h showed that on the basis of heat treatment of the A510 sample, the A400 sample annealed at 400 °C/10 h after quenching had precipitated many coarse second phases except Al_3_Zr and Al_7_Cu_2_Fe, which were easy to be enriched at the triangular grain boundary (as shown in Figure 4d). The results of XRD revealed that the coarse second phase was Al_7_Cu_4_Li (as shown in Figure 3). Therefore, the second phases of the A400 sample were the mixture of Al_3_Zr, Al_7_Cu_4_Li and Al_7_Cu_2_Fe phases.

Before and after heat treatments, the morphology and composition of the second phases in Al–Cu–Li alloy were identified using SEM and TEM. Since the insoluble Al_7_Cu_2_Fe remained stable throughout the heat treatment process [29], the difference of the second phases of the samples were mainly the difference of other second phases; the specific second phases differences were shown in Table 3.

### 3.2. Forming Results of Samples under Different Second Phases

**Figure 5:**
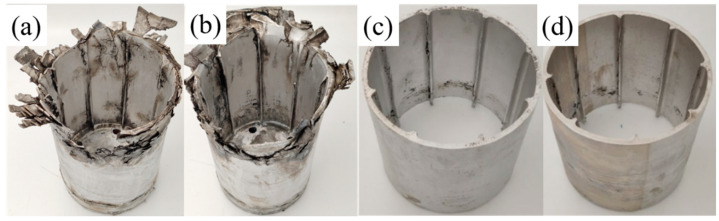
The results of the samples after composite spinning-extrusion forming; (**a**) PAN, (**b**) PA350, (**c**) PA510, (**d**) PA400.

After pretreatment, the samples were composite spinning-extrusion formed with the same forming process (as shown in Table 2);it was summarized in Figure 5. It could be clearly seen that A510 and A400 samples had good forming quality among all the samples. During the forming process of this work, the second phases of the samples after regulation were not dissolved [22], and the only forming variable was the regulated second phases. This meant that the microstructure of the AN and A350 samples with coarse phases, such as Al_2_Cu and Al_7_Cu_4_Li were not conducive to forming. However, the microstructures of the A510 and A400 samples dominated by Al_3_Zr dispersoid were suitable for forming.

### 3.3. Mechanical Properties of Cylindrical Parts under Different Second Phases

**Figure 6:**
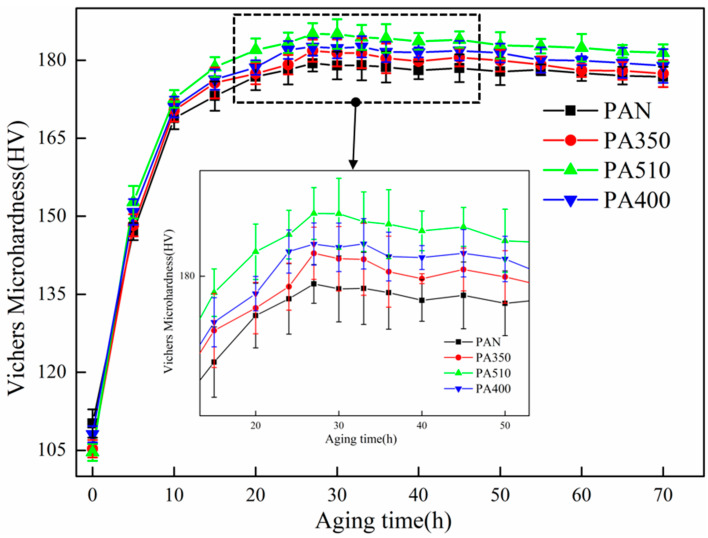
Aging hardening curve of the PAN, PA350, PA510 and PA400 samples.

**Figure 7:**
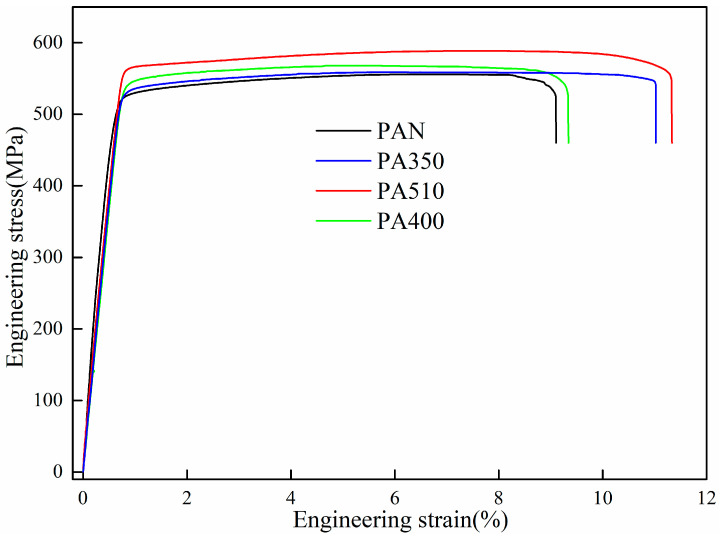
Stress–strain curve of the PAN, PA350, PA510 and PA400 samples at T8.

Figure 6 shows the aging hardening curves of the PAN, PA350, PA510 and PA400 samples. It could be seen that the average hardness of the four samples increases with the aging time, and decreases slowly after reaching the peak value at 27 h. The average hardness values of peak aging were 179.4, 181.8, 185.1 and 182.5 HV, respectively. Figure 7 depicts the stress–strain curve of the four samples at T8 and the relevant values of ultimate tensile strength, yield strength, elongation were presented in Table 4. As shown in Figure 7, the lowest ultimate tensile strength was found in the PAN sample (555 MPa) as compared with the PA400 (567 MPa) and the PA510 samples (588 MPa). Furthermore, the ultimate tensile strength of the PA350 sample was extremely similar to the PAN sample, while the elongation of the PA350 sample (11%) was slightly better than the PAN sample (9.1%) and the PA400 sample (9.3%). It was remarkable that the PA510 sample showed the highest tensile strength (588 MPa) and elongation (11.3%).

## 4. Discussion

### 4.1. Effect of the Second Phases on the Composite Spinning-Extrusion of Al–Cu–Li Alloy

**Figure 8:**
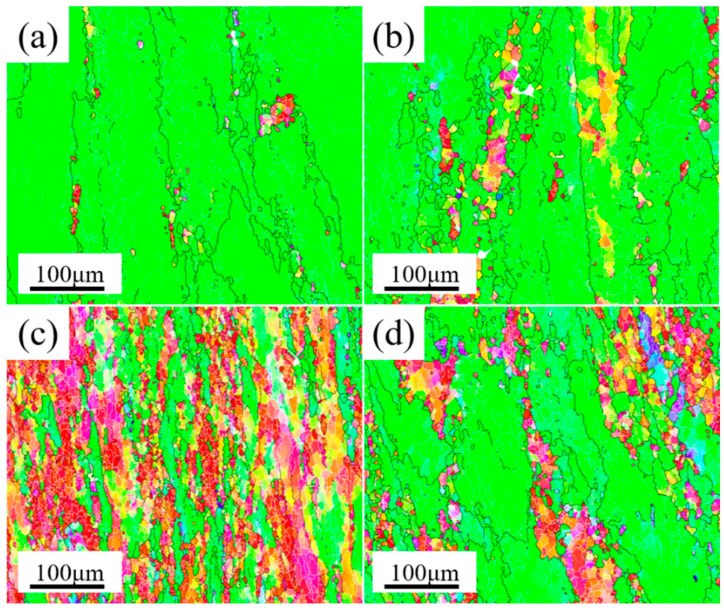
EBSD IPF maps of the four samples after composite spinning-extrusion forming; (**a**) PAN, (**b**) PA350, (**c**) PA510, (**d**) PA400.

The second phase was a direct reflection of the properties of Al–Cu–Li alloy, which not only had an obvious effect on the microstructure of Al–Cu–Li alloy, but also directly affected the forming of cylindrical parts. By comparing the forming quality of the samples formed via composite spinning-extrusion (as shown in Figure 5), it was found that the forming quality of the PA510 and PA400 samples were better. The macroscopic properties of the Al–Cu–Li alloy depended on the microstructures, which indicated that the microstructures formed during the forming process obviously affected the forming quality. Therefore, we conducted EBSD characterization of the microstructure of the four samples after forming, and the results were shown in Figure 8. Low angle grain boundary (LAGBs, defined as 2~15°) are represented by white lines and high angle grain boundary (HAGBs, defined as above 15°) are represented by black lines. It could be clearly seen that the dynamic recrystallization of the four samples occurred in different degrees after forming, and recrystallized grains were formed near the forming axial grains. However, the grains of the PAN and PA350 samples were elongated along the forming axis, the grain sizes (about 80 μm) were larger and the orientation consistency was enhanced. In addition, compared with the PAN and PA350 samples, the PA510 and PA400 samples had weaker grain orientation and smaller grain size (about 40 μm).

**Figure 9:**
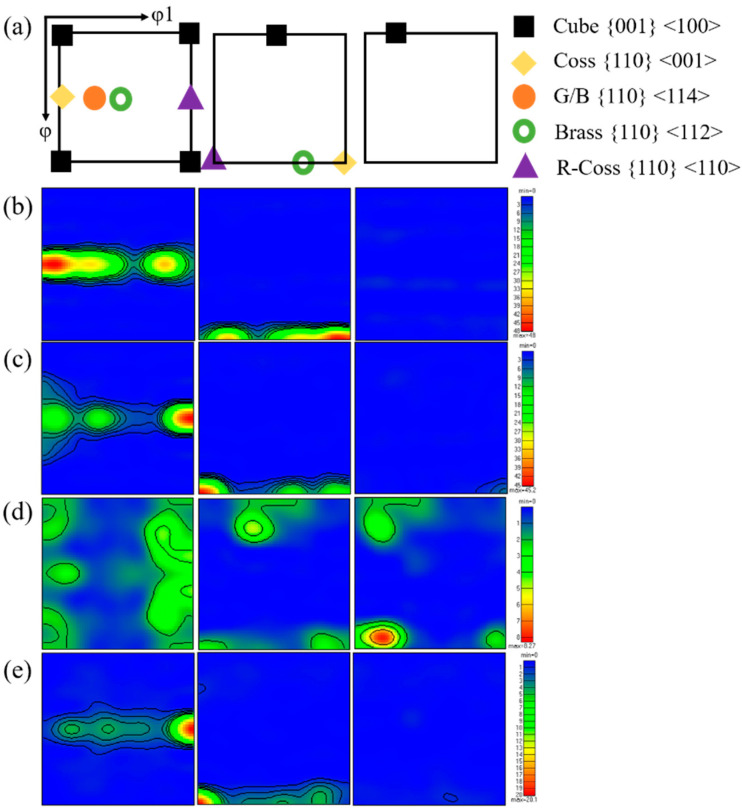
φ2 = 0, 45, and 65° sections of orientation distribution functions (ODF) maps of the samples; (**a**) Marked locations of texture components, (**b**) PAN, (**c**) PA350, (**d**) PA510, (**e**) PA400.

In general, Al–Cu–Li had high anisotropy in strength, and its strong texture was easy to form in the hot forming process [30]. In this paper, as a complex strong rheological forming process, the composite spinning-extrusion forming had higher sensitivity to the forming window of Al–Cu–Li alloy. Figure 9 compared the orientation distribution functions (ODF) of the four samples (φ2 = 0, 45, and 65°). Figure 9a showed the position labeling of major texture components in ODF. The results showed that coss and R-coss were the main textures of the four samples. However, the textural strength of the PA510 (8) and PA400 (20) samples were obviously less than that of the PAN (48) and PA350 (45) samples. In particular, the PA510 sample had weak texture strength, uniform grain growth in all orientations, and the best forming quality (as shown in Figure 5). By comparing the second phases of the four samples (as shown in Table 3), it could be seen that the difference between the first two samples (AN and A350) and the last two samples (A510 and A400) were whether the second phases contained Al_3_Zr dispersoid or not. In addition, the PA510 and PA400 samples exhibited weaker grain orientation and smaller grain size (as shown in Figure 8c,d). Al_3_Zr dispersoid had the obvious effect of nailing grain boundary movement, and its dispersoid distribution was considered to be a good microstructure [19]. The uniform distribution of Al_3_Zr dispersoid in the PA510 sample could effectively inhibit the abnormal grain growth along the deformation direction, and thus made the grain structure uniformly fine. The PAN and PA350 samples without Al_3_Zr dispersoid formed strong deformation texture during the forming process. It was obvious that the PAN and PA350 samples dominated by these strong deformation textures must exhibit poorer formability under the action of strong forming rheology. As seen from the results of Figure 5, a large number of cracks and folding deformation gathered at the end of the PAN and PA350 samples. These results showed that different second phases affected the texture strength and formability of Al–Cu–Li alloy during composite spinning-extrusion forming.

### 4.2. Effect of the Second Phases on the Mechanical Properties of Al–Cu–Li Alloy Cylindrical Parts

#### 4.2.1. Strength

**Figure 10:**
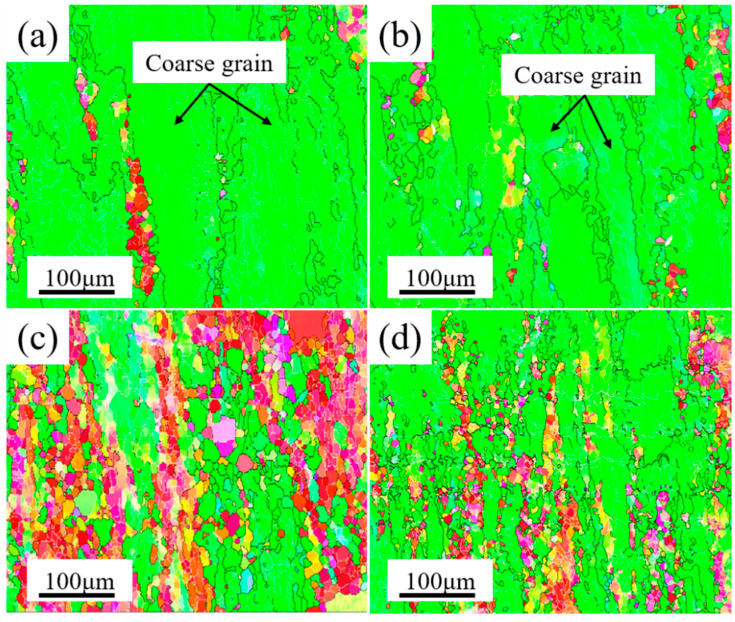
EBSD IPF maps of the four samples at T8; (**a**) PAN, (**b**) PA350, (**c**) PA510, (**d**) PA400.

The strengthening mechanism of Al–Cu–Li alloy included solid solution strengthening, fine crystal strengthening, second phases particle strengthening and work hardening [31]. Due to the same chemical composition, composite spinning-extrusion forming process, solution treatment and T8 heat treatment of the four samples, the strength difference at T8 were mainly due to the precipitation of aging strengthened phase and the contribution of grain refinement. As seen in Figure 7, compared with the PAN sample, the tensile strength of the PA510 sample increased from 555 MPa to 588 MPa. The results showed that the dissolution of coarse second phase particles and the precipitation of Al_3_Zr dispersion in blank were conducive to the improvement of mechanical properties of the cylinder. In addition, compared with the PA510 sample, the tensile strength of the PA400 sample decreased from 588 MPa to 567 MPa, and the elongation decreased from 11.3% to 9.3%, which indicated that the Al_7_Cu_4_Li phase particles had an adverse effect on the strength and plasticity of the cylindrical part. In order to explain these changes, the microstructures of the four samples at T8 were characterized. Figure 10 showed the grain structure of the four samples at T8. The results revealed that after T8 heat treatment, the four samples had further recovery and static recrystallization, and some new fine grains continued to be formed. However, the grains of the PAN and PA350 samples still showed strong orientation consistency, and had the tendency to continue growing along the direction of deformation. The average grain sizes of the four samples were calculated using the area-weighted method, and the average grain sizes were 97, 90, 45 and 50 μm, respectively. Compared with the average grain size of the four samples after forming, the average grain size after T8 heat treatment grew under the static recrystallization process. However, the grains of the PA510 and PA400 samples with Al_3_Zr dispersoid remained fine and uniform, while the PAN and PA350 samples were dominated by coarse grains. This further indicated that Al_3_Zr dispersoid can inhibit abnormal grain growth.

**Figure 11:**
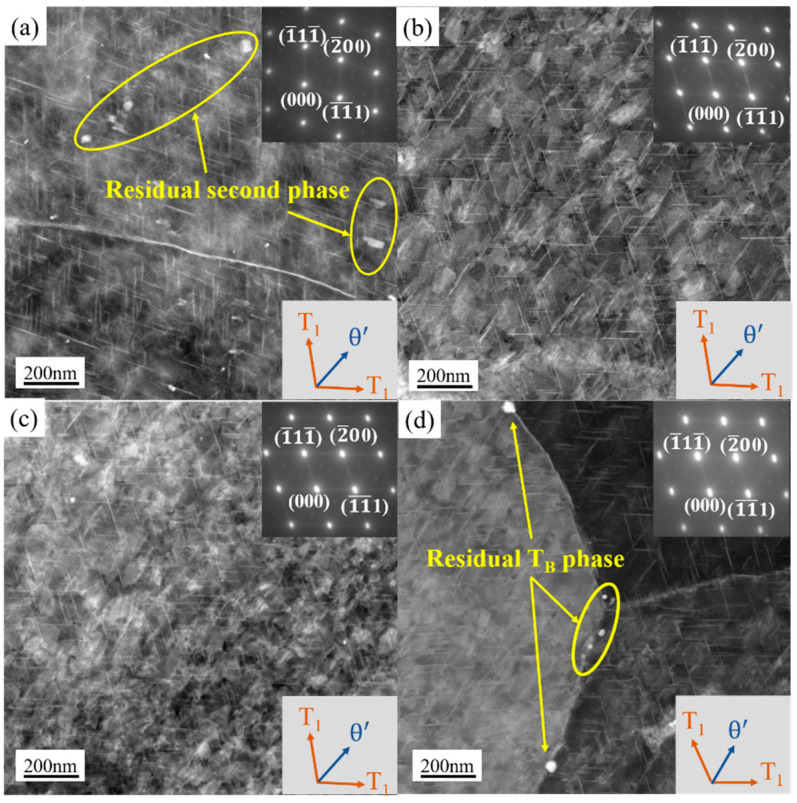
STEM maps of the four samples ([110] _Al_ zone axis) taken at T8; (**a**) PAN, (**b**) PA350, (**c**) PA510, (**d**) PA400.

**Figure 12:**
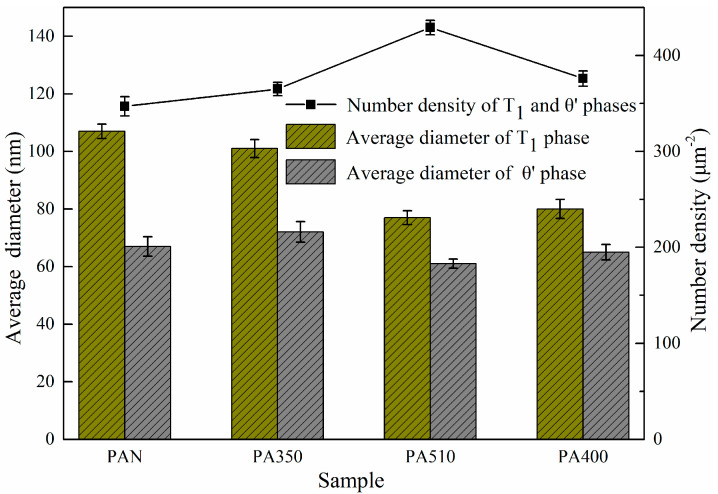
Average diameter and number density of T_1_ and θ’ phases.

Figure 11 highlighted the STEM results of the four samples at T8. The mean diameters and number densities of T_1_ and θ’ phases were calculated according to the HRTEM maps in Figure 12. The mean diameter measurement and quantity density calculation methods are the same as those in references [32,33]. The results showed that the average diameter of θ’ phase of the four samples were about 65 nm. However, the diameter of T_1_ phase of the PA510 and PA400 samples were about 77 nm on average, which were significantly smaller than 105 nm of the PAN and PA350 samples. The quantity density relationship between T_1_ and θ’ phases of the four samples were PA510 > PA400 > PA350 > PAN, which were basically consistent with the result of strength relationship in Figure 7.

It could be seen from Figure 11a,d that except in the T_1_ and θ’ phases, there were still some undissolved second phases in the PAN and PA400 samples containing a large amount of second phases in the blank. Both T_1_ and θ’ phases were Cu-rich strengthening phases. Compared with the other parts, these undissolved second phases occupy the Cu atoms required for the T_1_ and θ’ phases to some extent. In addition, the average grain size of the PAN sample was about twice of the PA510 sample. Therefore, the grain strengthening effect of the PAN sample was significantly lower than that of the PA510 sample. Although the average grain size of the PA400 sample was similar to that of the PA510 sample, the T_1_ phase number density was lower. Therefore, the ultimate tensile strength of the PAN and PA400 samples were significantly lower than that of the PA510 sample. As seen in Figure 11b, in addition to the T_1_ phase, many θ’ phase was precipitated in the PA350 sample. Because the T_1_ phase habitus plane was located right on the preferred slip plane of stretching and has a particularly high aspect ratio, the T_1_ phase could provide more significant reinforcement than the θ’ phase [4]. The precipitation and growth of θ’ phase will consume solute atoms in a short time, and inhibit the precipitation of T_1_ phase. In addition, compared with other parts, the average grain size of the PAN and PA350 samples were larger, and the contribution of fine grain strengthening were small. Therefore, the strength of the PA350 sample with low T_1_ phase number density was significantly lower than that of the PA510 sample.

**Figure 13:**
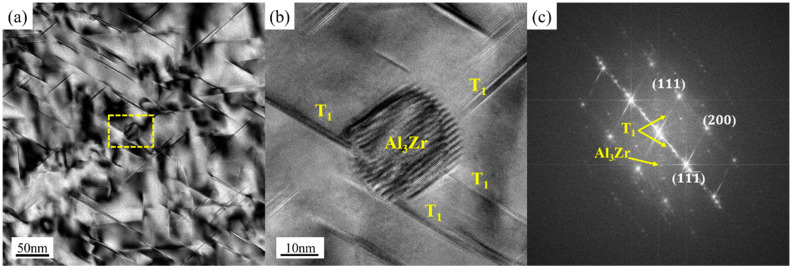
Bright field TEM, High-Resolution TEM and corresponding Fast Fourier Transform images of Al_3_Zr along [110] _Al_ direction of the PA510 sample at T8; (**a**) Bright field TEM, (**b**) High-Resolution TEM, (**c**) Fast Fourier Transform.

As seen from the results in Figure 11c and Figure 12, T_1_ phase of the PA510 sample was small and dense, achieving the highest strength. In order to know the origin of this phenomenon, more detailed observations were made on the PA510 sample, and the results were shown in Figure 13. Figure 13b,c showed the high-resolution and Fast Fourier transform images corresponding to the Al_3_Zr dispersoid, respectively. Multiple T_1_ phases were observed at the Al_3_Zr dispersoid interface. In the figure, the Al_3_Zr dispersoid was irregularly elliptic and semi-congruent with the matrix. The semi-coherent Al_3_Zr dispersoid has high interfacial energy with the matrix, which could provide higher nucleation driving force for the T_1_ phase and promote its growth. Therefore, the PA510 sample had the highest T_1_ phase number density. This was consistent with Araullo-Peters et al. [34] who confirmed that Al_3_Zr dispersoid could promote T_1_ phase growth. The compact distribution of fine precipitated phase was a typical structure of precipitated strengthening effect. In addition, the PA510 sample had the smallest average grain size and uniform grain orientation distribution. Therefore, compared with other samples, the PA510 sample exhibited excellent ultimate tensile strength.

#### 4.2.2. Elongation

**Figure 14:**
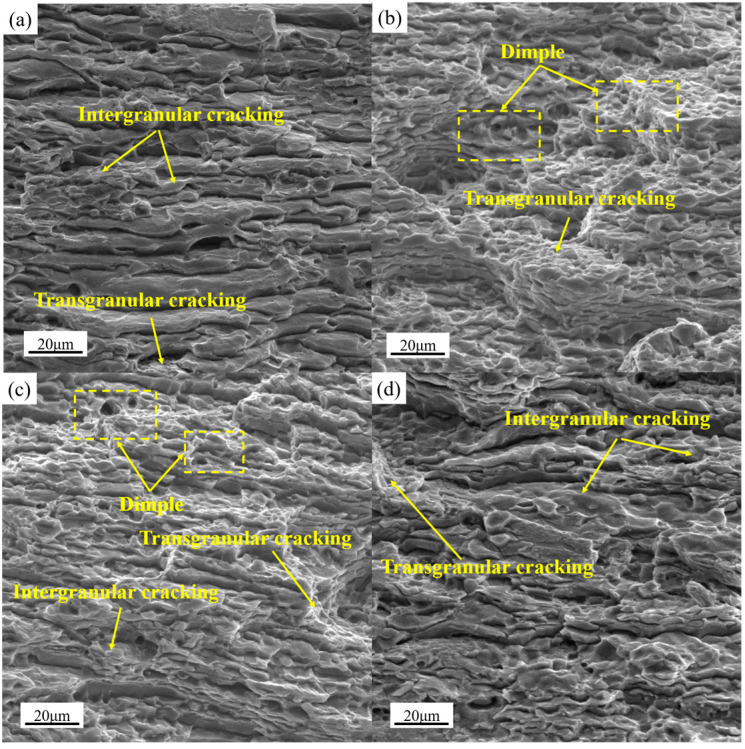
Fracture morphologies of the four samples at T8; (**a**) PAN, (**b**) PA350, (**c**) PA510, (**d**) PA400.

Figure 14 showed the SEM image of the tensile fractures of the four samples at room temperature at T8. It was found that the fracture morphologies in the all parts were mixed modes of transgranular and intergranular fracture. The fracture mode of the PA350 and PA510 samples were mainly transgranular fracture, and the PAN and PA400 samples were mainly intergranular fracture. Figure 14b,c showed the similar fracture morphologies of the PA350 and PA510 samples with more dimples, which implied better plasticity. However, the morphologies shown in Figure 14a,d were mainly smooth sections left by intergranular fracture, and the number of dimples were small and shallow, which indicated the poor plasticity of the PAN and PA400 samples. The poor plasticity of the PAN and PA400 samples may be due to the stress concentration caused by coarse second phases particles and grain boundary T_B_ phase [20,21]. Therefore, in order to ensure the property of Al–Cu–Li alloy cylindrical parts, the coarse second phases particles should be dissolved as much as possible and avoid precipitation at the grain boundary.

## 5. Conclusions

In this work, the effects of four typical second phases on the composite spinning-extrusion forming of Al–Cu–Li alloy were studied. In addition, the correlation between the second phases and the final mechanical properties of the cylinder part were further revealed. The main conclusions are as follows.

(1) The different second phases of Al–Cu–Li alloy can be adjusted via proper preheating treatment. The initial second phases of spray-formed Al–Cu–Li alloy is a mixture of coarse residual phases, such as Al_2_Cu, Al_7_Cu_4_Li and Al_7_Cu_2_Fe. Al_2_Cu and Al_7_Cu_4_Li can be dissolved during preheating at 510 °C/4 h. After quenching, the second phases mainly composed of θ’ phase and T_B_ phase can be obtained by annealing at 350 °C/2 h and 400 °C/10 h, respectively. Al_3_Zr dispersoid can be precipitated in large quantities under the condition of slow slope heating (0.85 °C/min, 510 °C/12 h).
(2) Dissolving the residual Al_7_Cu_4_Li and Al_2_Cu and precipitating Al_3_Zr dispersoid in the blank can effectively weaken the orientation consistency of the cylindrical parts and improve the quality of composite spinning-extrusion forming. After preheating treatment, the blank is well formed. At the same time, the grain distribution is more uniform and the average grain size is refined from about 80 μm to 40 μm.
(3) Fine Al_3_Zr dispersoid can effectively improve the final mechanical properties of composite spinning-extrusion cylindrical parts. Meanwhile, fine Al_3_Zr dispersoid can inhibit the abnormal growth of recrystallized grains during heat treatment and promote the precipitation of fine and dense T_1_ phase during artificial aging. After regulation, the average grain size of the cylindrical parts is refined from about 90 μm to about 45 μm, and the average diameter of T_1_ phase is refined from 107 nm to 77 nm. In addition, the ultimate tensile strength, yield strength and elongation of cylindrical parts are increased from 555 MPa to 588 MPa, 530 MPa to 564 MPa, and 9.1% to 11%, respectively.

## Figures and Tables

**Table 1:** Chemical composition of Al–Cu–Li alloy (wt%).

Cu	Li	Mg	Ag	Zr	Fe	Si	Zn	Mn	Ti	Al
3.77	1.16	0.46	0.31	0.13	0.08	0.06	0.008	0.001	0.001	Bal

**Table 2:** The main process parameters of composite spinning-extrusion forming.

Blank Dimensions (mm)	Forming Temperature T (°C)	Mandrel Speed n (r/min)	Feed Rate f (mm/r)	Reduction Rate ψ (%)
φ200 × 20	300	140	1.07	82.5

**Table 3:** The difference of the second phases of the samples.

Samples	Difference of the Second Phases
AN	The mixture of Al_2_Cu and Al_7_Cu_4_Li phases
A350	Mainly based on θ’ phase
A510	Uniform distribution of Al_3_Zr dispersoid
A400	The mixture of Al_3_Zr and Al_7_Cu_4_Li phases

**Table 4:** Ultimate tensile strength (UTS), yield stress (YS), elongation (EI) of the cylindrical parts with ribs.

Parts	UTS/MPa	YS/MPa	EI/%
PAN	555 ± 3	528 ± 4	9.1 ± 0.3
PA350	560 ± 5	530 ± 12	11.0 ± 0.4
PA510	588 ± 7	564 ± 8	11.3 ± 0.5
PA400	568 ± 4	543 ± 11	9.3 ± 0.2

## Data Availability

The data that support the findings of this study are available from the corresponding author, upon reasonable request.
